# Molecular analysis of the *BRCA1* and *BRCA2* genes in 32 breast and/or ovarian cancer Spanish families

**DOI:** 10.1054/bjoc.1999.1089

**Published:** 2000-03-06

**Authors:** A Osorio, A Barroso, B Martínez, A Cebrián, J M San Román, F Lobo, M Robledo, J Benítez

**Affiliations:** Department of Genetics, Service of Breast and Neck Surgery, Service of Oncology, Fundación Jiménez Díaz, Avenida Reyes Católicos 2, Madrid, Spain

**Keywords:** BRCA1, BRCA2, mutation, ovarian cancer, male breast cancer

## Abstract

It is estimated that about 5–10% of breast cancer cases may be due to inherited predisposition. Until now, two main susceptibility genes have been identified: *BRCA1* and *BRCA2*. The first linkage and mutational studies suggested that mutations in these two genes would account for the majority of high-risk breast cancer families, but recent studies show how the proportion of families due to *BRCA1* or *BRCA2* mutations strongly depends on the population and the types of family analyzed. It is now clear that, in the context of families with a modest cancer profile, which are the most commonly found in the clinical practice, the percentage of mutations found is much lower than that suggested by the first studies. In the present study, we analyze a group of 32 Spanish families, which contatined at least three cases of female breast cancer (at least one of them diagnosed before the age of 50 years), for the presence of mutations in the *BRCA* genes. The total proportion of mutations was low (25%), although the percentage of mutations in the *BRCA1* and *BRCA2* genes was higher, considering the breast and ovarian cancer families and the male breast cancer families respectively. Our results are in agreement with the idea that a great proportion of moderate-risk cancer families could be due to low penetrance susceptibility genes distinct from *BRCA1* or *BRCA2*. © 2000 Cancer Research Campaign


*British Journal of Cancer* (2000) **82**(7), 1266–1270
© 2000 Cancer Research Campaign
DOI: 10.1054/ bjoc.1999.1089, available online at http://www.idealibrary.com on IDEAL®

### Molecular analysis of the *BRCA1* and *BRCA2* genes in 32 breast and/or ovarian cancer Spanish families

**A Osorio[1], A Barroso[1], B Martínez[1], A Cebrián[1], JM San Román[2], F Lobo[3], M Robledo[1] and J Benítez[1]**

[1]Department of Genetics, [2]Service of Breast and Neck Surgery and [3]Service of Oncology, Fundación Jiménez Díaz, Avenida Reyes Católicos 2, Madrid, Spain

**Summary** It is estimated that about 5–10% of breast cancer cases may be due to inherited predisposition. Until now, two main susceptibility genes have been identified: *BRCA1* and *BRCA2*. The first linkage and mutational studies suggested that mutations in these two genes would account for the majority of high-risk breast cancer families, but recent studies show how the proportion of families due to *BRCA1* or *BRCA2* mutations strongly depends on the population and the types of family analyzed. It is now clear that, in the context of families with a modest cancer profile, which are the most commonly found in the clinical practice, the percentage of mutations found is much lower than that suggested by the first studies. In the present study, we analyze a group of 32 Spanish families, which contatined at least three cases of female breast cancer (at least one of them diagnosed before the age of 50 years), for the presence of mutations in the *BRCA* genes. The total proportion of mutations was low (25%), although the percentage of mutations in the *BRCA1* and *BRCA2* genes was higher, considering the breast and ovarian cancer families and the male breast cancer families respectively. Our results are in agreement with the idea that a great proportion of moderate-risk cancer families could be due to low penetrance susceptibility genes distinct from *BRCA1* or *BRCA2*.
© 2000 Cancer Research Campaign

**Keywords:** BRCA1; BRCA2; mutation; ovarian cancer; male breast cancer

Breast cancer is the most common malignancy among women in developed countries. A family history of breast and/or ovarian cancer is one of the main risk factors for the development of the disease (Lynch et al, 1981). It is estimated that about 5–10% of breast cancer cases may be due to inherited predisposition but the exact number of predisposing genes is unknown. In the context of high-risk families, epidemiological studies have provided evidence of at least two susceptibility genes: *BRCA1* (17q21) (Miki et al, 1994) and *BRCA2* (13q12) (Wooster et al, 1995). Early estimates suggested that *BRCA1* would be responsible for 45% of site-specific breast cancer families and the majority of breast and ovarian cancer families (Easton et al, 1993). These first studies also suggested that *BRCA2* might be responsible for 25–35% of site-specific breast cancer and would be implicated in the majority of male breast cancer families (Stratton et al, 1994). However, recent data show that these percentages could have been over-estimated and that the proportion of families with mutations in *BRCA1* or *BRCA2* strongly depends on the population analysed (Szabo and King, 1997) and the specific characteristics of the families selected (Schubert et al, 1997; Serova et al, 1997; Ford et al, 1998; Malone et al, 1998). These studies suggest that *BRCA1* could account for only about 15% of inherited breast cancer and about 45% of families with breast and ovarian cancer (Couch et al, 1997; Serova et al, 1997; Shattuck-Eidens et al, 1997).

In the present study we analyzed a group of 32 Spanish families with three of more cases of women affected with breast cancer, at least one of them diagnosed before the age of 50. The objective of the study was to establish the proportion of cases attributable to mutations in the *BRCA1* or *BRCA2* genes in our population and to contribute to the general knowledge of the proportion of mutations found in families with a modest cancer profile, which comprise the majority of the families seeking genetic advice. Although the percentage of mutations was low (25%), striking differences were observed when families were classified attending to the presence of ovarian cancer or male breast cancer. These results are useful to establish a guideline in the development of mutation screening for the *BRCA* genes.

*Received 16 June 1999*
*Revised 11 October 1999*
*Accepted 2 November 1999*

*Correspondence to:* A Osorio

### PATIENTS AND METHODS

### Breast/ovarian cancer families

Thirty-two breast and/or ovarian cancer families were selected from women who underwent surgery in Fundación Jiménez Díaz (Madrid) in the last 7 years. These families can be considered representative of the Spanish population as a whole, as they are ascertained in every area of the country. The selection criteria were as follows:

1. Families with three or more cases of women affected with breast cancer and at least one of them diagnosed before the age of 50 (27 cases)
2. Families with three or more cases of women affected with breast cancer and at least one case of male breast cancer diagnosed at any age (five cases).

### Mutation detection

We performed a molecular analysis of the complete coding sequence and exon–intron boundaries of the *BRCA1* and *BRCA2*

**Table 1a** *BRCA1* mutations

| Family | Female breast cancer | Average age | Ovarian cancer | BRCA1 mutation | Exon | Codon | Effect |
|---|---|---|---|---|---|---|---|
| M36 | 8 | 42.0 | – | 5272–1–G/A | Intron 18 | – | Splicing |
| M72 | 4 (2B)[a] | 38.6 | 3 | 5236GGA/GAA | 18 | 1706 | G1706E |
| M73 | 2 | 43.0 | 4 | 236delAG | 3 | 39 | Ter39 |

[a]B = bilateral breast cancer.

**Table 1b** *BRCA2* mutations

| Family | Female breast cancer | Average age | Male breast cancer | Other cancers[b] | BRCA2 mutation | Exon | Codon | Effect |
|---|---|---|---|---|---|---|---|---|
| M21 | 4(2B) | 44.5 | – | Pan, Hep, Lung, Int | DelAAAC | 11 | 936 | Ter956 |
| M22 | 4(3B) | 44.7 | 3 | Ov, Leu, Ut, Osteo, St | Del5 | 23 | 3009 | Ter3015 |
| M24[a] | 1 | 49.0 | 1 | Ov (3), NHL, St | DelAA | 14 | 2370 | Ter2390 |
| M45 | 11 | 47.7 | 1 | Ov | DelAA | 25 | 3104 | Ter3109 |
| M59 | 4 | 38.0 | – | Prostate | delAAAC | 11 | 936 | Ter956 |

[a]Five paternal relatives affected by breast cancer at unknown ages and four relatives affected by other cancers. [b]Pan = pancreatic cancer, Hep = hepatic cancer, Int = intestine cancer, Leu = leukaemia, Ut = uterine cancer, Osteo = osteosarcoma, NHL = non-Hodgkin's lymphoma, St = stomach cancer, Ov = ovarian cancer.

genes. For this study we used the PTT (protein truncation test), SSCP (single-strand conformation polymorphism) and direct sequencing methods.

### *PTT*

We used this method for the analysis of exon 11 of the *BRCA1* gene and exons 10 and 11 of the *BRCA2* gene. Exons were amplified by polymerase chain reaction (PCR) in overlapping fragments of 1000–2000 bp using primers previously described (Hogervost et al, 1995; BIC website), which contained an T7 promoter, a eukariotic translation initiation sequence and gene-specific sequences. The PCR conditions were the following: 250 ng of genomic DNA; 10 × PCR buffer (Boehringer Mannheim), a mix of 200 mM of dATP, dTTP, dGTP and dCTP; 40 pmol each primer and 2.5 U of *Taq* polymerase (Boehringer Mannheim). Approximately 1 μg of the PCR product was 'in vitro' transcribed and translated using the TnT[R] T7 Quick Coupled Transcription/Translation System from Promega. The proteins were labelled with biotinilated lysine (Promega) and electrophoresed in a 12.5% polyacrylamide-sodium dodecyl sulphate (SDS) gel. The detection was performed using the 'Transcend™ Chemiluminescent Non-Radioactive Translation' from Promega.

### *SSCP*

SSCP was used for the analysis of exons 2–10 and 12–24 of the *BRCA1* gene, and 2–10 and 12–27 of the *BRCA2* gene using primers previously described (Simard et al, 1994; Stratton et al, personal communication). Fragments of 200–300 bp were amplified under standard PCR conditions containing 100 ng genomic DNA, 10 × PCR buffer (Boehringer Mannheim), a mix of 200 μM of dATP, dTTP, dGTP and 10 mM of dCTP, 1 μl of 10–25 pmol each primer, 1.75 U of *Taq* polymerase (Boehringer Mannheim) and 0.5 μCi $^{32}P$ dCTP (INC). In some cases, 2% of dimethyl sulphoxide (DMSO) and 0.1% of Triton X-100 were added to improve the amplification. Amplification was performed in a Perkin-Elmer Cetus 2400 thermocycler. PCR amplification conditions were as follows: 35 cycles of denaturing at 94°C for 45 s, annealing for 30 or 45 s and in some cases extension at 72° for 30 s. Reaction products were run in a SSCP electrophoresis gel under two conditions (room temperature, 10 W; 4°C, 50 W). Suspected mutants were identified as having a mobility shift.

### *Direct sequencing*

All the variants found by both methods were sequenced to verify the mutation. The DNA isolated from mutant allele carriers was amplified by PCR and the products were purified using an EZNA Cycle-pure kit (Labclinic). The purified DNA was subjected to cycle sequencing using an automated fluorescence-based cycle sequencer (Abi Prism 310, Perkin-Elmer) and dye terminator system.

### RESULTS

### *BRCA1* and *BRCA2* mutations

Only three of the 22 site-specific female breast cancer families were found to harbour mutations, one in the *BRCA1* gene and two in the *BRCA2* gene (Table 1). The *BRCA1* mutation identified was a splice-site mutation previously described. The two families with a *BRCA2* mutation showed the same alteration, 936delAAAC in exon 11, which is considered one of the recurrent mutations in the *BRCA2* gene (Neuhausen et al, 1998).

Two of five families with breast and ovarian cancer and no males affected showed mutations in the *BRCA1* gene. One was a frameshift mutation in exon 3, 39delTG, not previously described. The second alteration was an amino acid substitution in exon 18 that was classified as a missense mutation, as it was found in two affected sisters from the same family, but it was not detected in 200 control chromosomes. This alteration would be expected to be significant, since it results in the substitution of a small hydrophobic amino acid by a hydrophilic-charged one, and it is localized in the COOH terminal region of the gene which is

© 2000 Cancer Research Campaign 

**Table 2a** Polymorphisms found in the BRCA1 gene

| Variant[a] | Exon | Codon | Amin acid change | Frequency[b] |
|---|---|---|---|---|
| IVS8-57delT | Intron 8 | – | – | 16/54 |
| 4447T/C | 13 | 1436 | Ser-Ser | 72/180 |
| 4956A/G | 16 | 1613 | Ser-Gly | 6/50 |
| 5272+66G/A | Intron 18 | – | – | 17/54 |

[a]The number indicates the nucleotide in which the alteration ocurrs. All the variants have been previously described. [b]The frequency is obtained by the number of chromosomes which showed the variant/total number of chromosomes analysed in our breast cancer population.

**Table 2b** Polymorphisms found in the BRCA2 gene

| Variant | Exon | Codon | Amino acid change | Frequency | Frequency in control population |
|---|---|---|---|---|---|
| CAG/CAA | 2 | – | – | 3/26 | – |
| 909+56C/T | Intron 8 | – | – | 2/26 | – |
| AAT/CAT | 10 | 289 | Asn-His | 1/32 | 4/112 |
| TCA/TCG | 10 | 470 | Ser-Ser | 1/24 | |
| AAA/AAG[a] | 11 | 1132 | Lys-Lys | 2/22 | 19/80 |
| AAA/GAA[a] | 11 | 1286 | Lys-Glu | 9/26 | 3/116 |
| AGC/AGT | 11 | 1528 | Ser-Ser | 1/26 | – |
| ACG/ATG | 11 | 1915 | Thr-Met | 1/26 | – |
| TCA/TCG | 14 | 2414 | Ser-Ser | 3/24 | – |
| AAT/AGT[b] | 4 | 108 | Asn/Ser | 1/26 | 1/200 |
| ACC/GCC[b] | 27 | 3349 | Thr/Ala | 1/26 | 1/200 |

[a]Polymorphisms not previously described. [b]Unknown significant variants.

**Table 3** Proportion of mutations in the *BRCA1* and *BRCA2* genes

| Group of families | No. of families | *BRCA1* mutations | *BRCA2* mutations | Total proportion of mutations |
|---|---|---|---|---|
| Breast site-specific cancer families | | | | |
| 3–5 cases | 18 | – | 11% (2) | 11% (2) |
| > 5 cases | 4 | 25% (1) | – | 25% (1) |
| Total | 22 | 4.5% (1) | 9% (2) | 13.5% (3) |
| Breast and ovarian cancer families | 5 | 40% (2) | – | 40% (2) |
| Male breast cancer families | 5 | – | 60% (3) | 60% (3) |
| All families | 32 | 9.3% (3) | 15.6% (5) | 25% (8) |

highly conserved and supposed to be functionally important (Abel et al, 1995).

Three of five families with male breast cancer (60%), revealed frameshift mutations in the *BRCA2* gene (Table 1). All the families with mutations also contained cases of ovarian cancer and in two of them, there were several members affected by other types of cancer.

Surprisingly, in the case of the *BRCA1* gene we did not detect any mutation in exon 11, which, to date, is supposed to accumulate a great proportion of the mutations identified in most of the studies. Our results are in agreement with those derived from other studies in Spanish population (Baiget et al, personal communication; Caldés et al, personal communication). In the case of *BRCA2*, the mutations identified were distributed between exon 11 and exon 27. We found unknown significance variants in the *BRCA2* gene in two families (Table 2): one of them (family 44), contained three cases of female breast cancer and four additional members of the family affected by tyroid, stomach, intestine and liver cancer, the second case (family 68) contained three cases of female breast cancer. We did not have enough data to accept or rule out the implication of these variants in the pathology of the disease in none of the cases. We also found a great number of polymorphisms in both genes (Table 2).

### Classification of families

Although the proportion of mutations was low considering all the families (25%), the percentages were strongly modified when we analysed different groups of families. We categorized the families, according to the number of breast cancer cases, the presence of ovarian cancer and the presence of male breast cancer (Table 3).

© 2000 Cancer Research Campaign

The lowest proportion of mutations was found in the site-specific female breast cancer families (13.5%). In the breast and ovarian cancer families the percentage of mutations was 40% and all of them were found in the *BRCA1* gene. In the case of male breast cancer families, we found 60% of mutations and all of them were found in the BRCA2 gene.

### DISCUSSION

Although there are some *BRCA1* and *BRCA2* mutational studies in the Spanish population, they are focused on women with sporadic or early onset breast cancer (Garcia-Patino et al, 1998) or they are limited to the analysis of possible recurrent mutations (Osorio et al, 1998; Díez et al, 1999). The present study is the first one in which the whole coding region of the *BRCA1* and *BRCA2* genes are analysed in a group of selected breast and/or ovarian cancer Spanish families. The proportion of mutations in the *BRCA1* or *BRCA2* genes found in our 32 families was low (25%). These results are in agreement with several recent studies that show how the percentage of mutations found in the *BRCA* genes is much lower than the predicted from the linkage studies. Most of the first linkage and mutational analysis of familial breast cancer suggested that mutations in the *BRCA1* and *BRCA2* genes together would account for most of the high-risk breast and/or ovarian cancer families (Easton et al, 1993). However, these studies were focused on rare families with an extremely large number of affected women. This and other studies evidence how the majority of families attending genetic counselling show a modest cancer profile and that it is important to establish the real implication of the *BRCA* genes in this group (Ford et al, 1998; Malone et al, 1998).

The lowest percentage of mutations, 13.5%, was found in the breast site-specific cancer families. Recent studies suggest that in this types of family, *BRCA1* mutations would account for only 10–20% of the cases and probably the same would be for *BRCA2* (Blackwood & Weber, 1998). These studies suggest that most of the families with five or fewer cases of women affected with breast cancer and without any case of ovarian or male breast cancer would be attributable to mutations in other low-penetrance susceptibility genes distinct from *BRCA1* and *BRCA2* (Schubert et al, 1997; Serova et al, 1997; Ford et al, 1998; Malone et al, 1998); the majority of our families contained 3–5 cases of breast cancer, which explains the low proportion of mutations found.

In the group of breast and ovarian cancer families, the percentage of mutations was 40% and all of them were found in the *BRCA1* gene. This proportion is identical to that found in the last studies, in which up to 45% of breast and ovarian cancer families turned out to be attributable to mutations in the *BRCA1* gene (Couch et al, 1997; Serova et al, 1997; Shattuck-Eidens et al, 1997). We did not find any relationship between the number of breast cancer cases in the family or the existence of bilateral breast cancer, and the presence of mutation, but this was probably due to the small number of families analysed.

Sixty per cent of the families with at least one case of male breast cancer harbour a mutation in the *BRCA2* gene. This percentage is high and similar to the found in other studies (Ford et al, 1998), and shows once again the relationship which exists between male breast cancer and mutations in the *BRCA2* gene.

We observed a number of tumours other than breast and ovary, in the *BRCA2* families. This is in agreement with previous studies that suggest that mutations in *BRCA2* confer an increased risk for different types of cancer. A recent work from the Breast Cancer Linkage Consortium shows that pancreatic and prostate cancers are the most frequent in *BRCA2* families. In our case, we found two families with one case each of prostate and pancreatic cancer, but all the rest of the tumours observed were of different types.

It is important to note that up to 30% of the mutations can be localized outside the coding region of the genes or consist of great deletions or rearragements not detectable by conventional techniques. On the other hand, we can find mutations that cause an amino acid substitution whose effect in the protein function is unknown. These variants cannot be considered as mutations but it is not possible to rule out their role in the development of the disease. We found unknown significance in variants in two of our families. Although all these factors can contribute to the low number of mutations detected, they are not supossed to strongly affect the percentages.

In summary, our study confirms once again that the proportion of mutations in the *BRCA1* and *BRCA2* genes in breast cancer families is low, making the genetic testing and counselling extremely difficult. However, the presence of at least one case of ovarian cancer or male breast cancer is a strong predictor for the presence of mutations in the *BRCA1* and *BRCA2* genes respectively, which makes these kinds of family specially eligible for the mutational studies.

### ACKNOWLEDGEMENTS

This study was partially supported by CICYT 96/0192 FIS 99/0140, CAM 081/0009/97. Ana Osorio is a fellow of Fundación Conchita Rábago.

### REFERENCES

Abel KJ, Xu J, Yin GY, Lyons RH, Meisler MH and Weber BL (1995) Mouse *BRCA1*: localization sequence analysis and identification of evolutionarily conserved domains. *Hum Mol Genet* **4**: 2265–2273

BIC: *www.nchgr.nih.gov/Intramural_research/Lab_transfer/Bic/guidelines.html*

Blackwood MA and Weber BL (1998) BRCA1 and BRCA2: from molecular genetics to clinical medicine. *J Clin Oncol* **16**: 1969–1977

Couch FJ, DeShano ML, Blackwood MA, Calzone K, Stopfer J, Campeau L, Ganguly A, Rebbeck T and Weber B (1997) BRCA1 mutations in women attending clinics that evaluate the risk of breast cancer. *N Engl J Med* **336**: 1409–1415

Díez O, Domènech M, Alonso MC, Brunet J, Sanz J, Cortes J, del Rio E and Baiget M (1998) Identification of the 185delAG BRCA1 mutation in a Spanish Gypsy population. *Hum Genet* 1998 **103**: 707–708

Díez O, Osorio A, Robledo M, Barroso A, Domènech M, Cortés J, Albertos J, Sanz J, Brunet J, San Román JM, Alonso MC, Baiget M and Benítez J (1999) Prevalence of BRCA1 and BRCA2 Jewish mutations in Spanish breast cancer patients. *Br J Cancer* **79**: 1302–1303

Easton DF, Bishop DT, Ford D and Crockford GP (1993) Genetic linkage analysis in familial breast and ovarian cancer: results from 214 families. The Breast Cancer Linkage Consortium. *Am J Hum Genet* **52**: 678–701

Ford D, Easton DF, Stratton M, Narod S, Goldgar D, Devilee P, Bishop DT, Weber B, Lenoir G, Chang-Claude J, Sobol H, Teare MD, Struewing J, Arason A, Scherneck S, Peto J, Rebbeck TR, Tonin P, Neuhausen S, Barkardottir R, Eyfjord J, Lynch H, Ponder BA, Gayther SA, Zelada-Hedman M and the Breast Cancer Linkage Consortium (1998) Genetic heterogeneity and penetrance analysis of the BRCA1 and BRCA2 genes in breast cancer families. The Breast Cancer Linkage Consortium. *Am J Hum Genet* **62**(3): 676–689

Garcia-Patino E, Gomendio B, Provencio M, Silva JM, Garcia JM, Espana P and Bonilla F (1998) Germ-line BRCA1 mutations in women with sporadic breast cancer: clinical correlations. *J Clin Oncol* **16**: 115–120

*© 2000 Cancer Research Campaign*

Hogervost FBL, Cornelis RS, Bout M, van Vliet M, Oosterwijk JC, Olmer R, Bakker B, Klijn JGM, Vasen HFA, Meijers-Heijboer H, Menko FH, Cornelisse CJ, den Dunnen JT, Devilee P and van Ommen G-JB (1995) Rapid detection of BRCA1 mutations by the protein truncation test. *Nat Genet* **10**: 208–212

Lynch HT, Fain PR, Golgar D, Albano WA, Mailliard JA and McKenna P (1981) Familial breast cancer and its recognition in an oncology clinic. *Cancer* **47**: 2730–2739

Malone KE, Daling JR, Thompson JD, O'Brien CA, Francisco LV and Ostrander EA (1998) BRCA1 mutations and breast cancer in the general population: analyses in women before age 35 years and in women before age 45 years with first-degree family history. *JAMA* **279**: 922–929

Miki Y, Swensen J, Shattuck-Eidens D, Futreal PA, Harshman K, Tavtigian S, Liu Q, Cochran C, Bennet LM, Ding W, Bell R, Rosenthal J, Hussey C, Tran T, McClure M, Frye C, Hattier T, Phelps R, Haugen-Strano A, Katcher H, Yakumo K, Gholami Z, Shaffer D, Stone S, Bayer S, Wray C, Bogden R, Dayananth P, Ward J, ToninP, Narod S, Brtistow PK, Norris FH, Helvering L, Morrison P, Rosteck P, Lai M, Barret C, Lewis C, Neuhausen S, Cannon-Albright L, Goldgar D, Wiseman R, Kamb A and Skolnik M (1994) A strong candidate for the breast and ovarian cancer susceptibility gene BRCA1. *Science* **266**: 66–71

Neuhausen SL, Godwin AK, Gershoni-Baruch R, Schubert E, Garber J, Stoppa-Lyonnet D, Olah E, Csokay B, Serova O, Lalloo F, Osorio A, Stratton M, Offit K, Boyd J, Caligo MA, Scott RJ, Schofield A, Teugels E, Schwab M, Cannon-Albright L, Bishop T, Easton D, Benitez J, King MC and Goldgar D (1998) Haplotype and phenotype analysis of nine recurrent BRCA2 mutations in 111 families: results of an international study. *Am J Hum Genet* **62**: 1381–1388

Osorio A, Robledo M, Albertos J, Diez O, Alonso C, Baiget M and Benitez J (1998) Molecular analysis of the six most recurrent mutations in the BRCA1 gene in 87 Spanish breast/ovarian cancer families. *Cancer Lett* **123**: 153–158

Schubert EL, Lee MK, Mefford HC, Argonza RH, Morrow JE, Hull J, Dann JL and King MC (1997) BRCA2 in American families with four or more cases of breast or ovarian cancer: recurrent and novel mutations, variable expression, penetrance, and the possibility of families whose cancer is not attributable to BRCA1 or BRCA2. *Am J Hum Genet* **60**: 1031–1040

Serova OM, Mazoyer S, Puget N, Dubois V, Tonin P, Shugart YY, Goldgar D, Narod SA, Lynch HT and Lenoir GM (1997) Mutations in BRCA1 and BRCA2 in breast cancer families: are there more breast cancer-susceptibility genes? *Am J Hum Genet* **60**: 486–495

Shattuck-Eidens D, Oliphant A, McClure M, Mc Bride C, Gupte J, Rubano T, Pruss D, Tavtigian SV, Teng DHF, Adey N, Staebell M, Gumpper K, Lundstrom R, Hulick M, Kelly M, Holmen J, Ligenfelter B, Manley S, Fujimura F, Luce M, Ward B, Cannon-Albright L, Steele L, Offit K, Giewski T, Norton L, Brown K, Schulz Ch, Hampel H, Schluger A, Giulotto E, Zoli W, Ravaioli A, Nevanlinna H, Pyrhonen S, Rowley P, Loader S, Osborne MP, Daly M, Tepler I, Weinstein PL, Scalia JL, Michaelson R, Scott RJ, Radice P, Pierotti MA, Garber JE, Isaaes C, Peshkin B, Lippman ME, Dosik MH, Caligo MA, Greenstein RM, Pilarski R, Weber B, Burgemeister R, Frank TS, Skolnick M and Thomas A (1997)

Simard J, Tonin P, Durocher F, Morgan K, Rommens J, Gingras S, Samson C, Lebanc J-F, Bélager C, Dion F, Liu Q, Skolnick M, Goldgar D, Shattuck-Eidens D, Labrie F and Narod S (1994) Common origin of BRCA1 mutations in Canadian breast and ovarian cancer families. *Nat Genet* **8**: 392–398

Stratton MR, Ford D, Neuhasen S, Seal S, Wooster R, Friedman LS, King MC, Egilsson V, Devilee P and McManus R (1994) Familial male breast cancer is not linked to the BRCA1 locus on chromosome 17q. *Nat Genet* **7**: 103–107

Szabo CI and King MC (1997) Population genetics of BRCA1 and BRCA2. *Am J Hum Genet* **60**: 1013–1020

The Breast Cancer Linkage Consortium (1999) Cancer risks in BRCA2 mutation carriers. *J Natl Cancer Inst* **91**: 1310–1316

Wooster R, Bignell G, Lancaster J, Swift S, Seal S, Mangion J, Collins N, Gregory S, Gumbs C, Micklem G, Barfoot R, Hamoudi R, Patel S, Rice C, Biggs P, Hashim Y, Smith A, Connor F, Arason A, Gudmundson J, Ficenec D, Kelsell D, Ford D, Tonin P, Bishop DT, Spurr NK, Ponder BAJ, Eeles R, Peto J, Devilee P, Cornelisee C, Lynch H, Narod S, Lenoir G, Egilsson V and Bjork MR (1995) Identification of the breast cancer susceptibility gene BRCA2. *Nature* **378**: 789–792

 *© 2000 Cancer Research Campaign*
